# Disease severity of the first COVID-19 wave in Germany using reporting data from the national notification system

**DOI:** 10.25646/7170

**Published:** 2020-11-18

**Authors:** Julia Schilling, Ann-Sophie Lehfeld, Dirk Schumacher, Alexander Ullrich, Michaela Diercke, Silke Buda, Walter Haas, RKI COVID-19 Study Group

**Affiliations:** 1 Robert Koch Institute, Berlin Department of Infectious Disease Epidemiology; 2 Federal Institute for Quality Assurance and Transparency in Healthcare (IQTIG), Berlin Unit for Medical Biometry and Statistics

**Keywords:** COVID-19, PANDEMIC, FIRST WAVE, GERMANY, SEVERITY OF DISEASE, NATIONAL NOTIFICATION SYSTEM

## Abstract

As of December 31, 2019, initial reports circulated internationally of an unusual cluster of pneumonia of unknown cause in China. By the end of January 2020, the virus affected Germany with the first case confirmed on January 27, 2020. Intensive contact tracing and infection control measures contained the first two clusters in the country. However, the dynamic of the first wave gained momentum as of March, and by mid-June 2020 over 190,000 laboratory-confirmed cases had been reported to the Robert Koch Institute. This article examines these cases as part of a retrospective descriptive analysis focused on disease severity. Most cases (80%) were mild and two thirds of the cases were younger than 60 years (median age: 50 years). Severe cases were primarily reported among men aged 60 or over who had at least one risk factor (particularly cardiovascular disease, diabetes, neurological disorders and/or lung diseases). Cases between the ages of 40 and 59 years had the longest interval between symptom onset and hospitalisation (median: six days) and – if admitted to an intensive care unit (ICU) – also the longest ICU stay (median: eleven days). This analysis provides valuable information about disease severity of COVID-19 and particularly affected groups.

## 1. Introduction

As of December 31, 2019, initial reports circulated internationally of an unusual cluster of pneumonia of unknown cause in China [1]. Initial investigations suggested an epidemiological link to Huanan Seafood Market in Wuhan (Hubei Province, China) and a zoonotic (animal) origin. A few days later, on January 9, 2020, the World Health Organization (WHO) officially confirmed that the previously unknown virus was a corona virus. Initially titled 2019-nCoV, the virus has now been categorised as Severe Acute Respiratory Syndrome Coronavirus 2 (SARS-CoV-2) and the disease as Coronavirus Disease-2019 (COVID-19) [2]. Due to the close link to Wuhan, human-to-human transmissions was assumed to be limited at that time. This assessment changed soon as the number of cases increased continuously in China and the epidemic began spreading to other countries.

By the end of January 2020, the virus affected Germany with the first case confirmed on January 27, 2020 [3]. This case was a 33-year-old man employed by a company in Bavaria. In the course of the investigation, a colleague from the Chinese branch of the company, who had travelled from China to facilitate workshops and meetings, was identified as the source of the infection [4, 5]. Finally, contract tracing and investigations at the local, regional and national level have been crucial measures to contain this first outbreak in Germany. A second cluster consisted of two cases that were among a group of individuals, who were repatriated from China. They were tested positive after their arrival and were subsequently hospitalised for isolation, whereas the other passengers, repeatedly tested negative, remained in quarantine for two weeks [3]. These first two outbreaks provided valuable information about the transmission of the novel virus in Germany. The dynamic of the first corona virus wave in Germany gained momentum in February, worsened at the beginning of March and was triggered by outbreaks related to carnival events and to individuals returning from ski resorts (especially from Italy and Austria) [6]. Extensive infection and control measures were implemented and contained this first wave until mid-June. The situation in Germany has been continually assessed using the Pandemic Influenza Severity Assessment by WHO (PISA) [7], considering transmission, disease severity and impact on the health system.

Based on reported data from the national notification system, the first COVID-19 wave in Germany was analysed focussing on disease severity as one part of the risk assessment and in order to prepare for the upcoming autumn/winter season.

## 2. Methods

The following descriptive, retrospective analysis is based on laboratory-confirmed cases with a SARS-CoV-2 infection notified to the local health authority in line with the Protection against Infection Act (Infektionsschutzgesetz, IfSG) and subsequently reported to the federal state authority and further to the national database of notifications at the Robert Koch Institute (RKI) [8]. All cases reported to the Robert Koch Institute up to October 26, 2020 were included in line with the case definition of the RKI for laboratory confirmed infections of SARS-CoV-2. Therefore only laboratory-confirmed cases (pathogen isolation, Nucleic acid detection) with SARS-CoV-2 infection, irrespective of clinical symptoms are reported [8].

A total of 437,866 COVID-19 cases had been reported to the RKI by 26 October 2020. Of these, 190,816 cases originated from January 2020 until mid-June 2020, or reporting week 25. This study only includes cases that provide data on age, hospital status and death (n=166,662 by reporting week 25). In addition, hospital admission and discharge dates were also required to calculate periods in context of hospitalisation. This information was only available in around half of all cases, with a continuous decrease in the number of cases that provided this information as of reporting week 20. Assessing disease severity based on current case numbers may lead to an underestimation of the proportions of severe and fatal cases. In order to assess disease severity regarding proportions of serious disease outcomes and fatal cases, only numerators and denominators should be used for periods where relatively reliable information is available for both parameters. Previous analyses have shown that completeness of hospitalisation data decreases continuously the closer the reporting date is to the date of the analysis. As we assume that missing data would not be provided in a timely manner and further corrections are not to be expected, the analysis of the first wave only includes cases that were reported by reporting week 20 (n=152,984).

**Info box:**

**Table:** 

Disease severity	Definition based on the notification software
**Mild**	Clinical data available, no pneumonia, no hospitalisation, not deceased
**Moderate**	Clinical data available, pneumonia, no hospitalisation, not deceased
**Severe**	Hospitalised (regardless of availability of clinical data, intensive care and death)
**Critical**	Hospitalised, intensive care (regardless of availability of clinical data and death) Fatalities (regardless of availability of clinical data and hospitalisation)

Furthermore, intervals were calculated using cases with only one hospital stay. Intervals relating to symptom onset considered only cases with symptom onset before hospital/intensive care unit (ICU) admission and before cut-off date (October 26, 2020). Calculations of the length of hospitalisation/ICU-stay only considered cases with information on admission and discharge dates before 26 October 2020. Date of death was used in lieu of a discharge date if this was not available.

Incidences were calculated using the standard German population as of 31 December 2019. Analyses were carried out with StataSE 15, Microsoft Excel 2010 and R (version 3.6.1).

In line with the premise of providing ‘two meaningful digits’, figures that are lower than ten are given to one decimal point, whereas whole numbers are provided in other cases.

A distinction is made between mild, moderate, severe and critical cases (Info box), but these distinctions are not mutually exclusive. These categories are based on the initial description of disease severity drawn up by the WHO [9] during the first joint mission that took place in China and a preliminary assessment of disease severity using the reported data from the national notification system [10].

Symptoms (e.g. cough, runny nose) and severity parameters (e.g. acute respiratory distress syndrome (ARDS) and ventilation) can be entered into the notification software under ‘clinical data’. The software provides for binary responses (‘yes’ or ‘no’), with the default set to ‘no’. However, an additional parameter – the general availability of clinical data (‘yes’, ‘no’) – was previously considered. This enabled a clearer differentiation to be made between responses and was used to evaluate mild and moderate cases and symptoms.

Fatal cases were defined as laboratory confirmed cases with a SARS-CoV-2 infection who were reported to have died due to COVID-19 (“died of”) as well as people who had underlying conditions (in addition to COVID-19). In the latter case, it is impossible to conclusively identify the cause of death, but COVID-19 is assumed to have contributed to the death (“died with”).

The findings set out here on risk factors are based on the risk factors that can be entered into the notification software; these include diseases of the cardiovascular system, diabetes, neurological disorders, lung diseases, kidney diseases, cancer, immunological disorders and liver diseases.

## 3. Results

**Figure 1:**
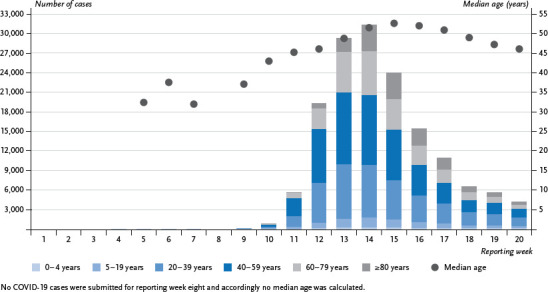
Number of reported COVID-19 cases in Germany by age group and median age over time, up to reporting week 20 (n=152,984) Source: COVID-19 cases reported to the RKI (data as of 26 October 2020, 00:00)

Once the first cases connected to a cluster in Bavaria and a group of people who had been repatriated to Germany became known at the end of January/beginning of February, the COVID-19 outbreak gained momentum. This was particularly the case at the beginning of March 2020, and, as of reporting week 10, the outbreak developed into Germany’s first wave of COVID-19 (Figure 1).

### 3.1 Demographic distribution and symptoms

Around 52% of the 152,984 cases that had been reported by reporting week 20 and which provided information about age, hospitalisation and death involved women. Whereas at the beginning of the wave, slightly more men had been affected than women, by reporting week 14, this ratio had reversed and the proportion of cases rose among women. The rate peaked in reporting week 15 at 57%. The majority of all cases (35%, n=53,392) were among 40- to 59-year-olds, followed by 20- to 39-year-olds (28%, n=42,801) and 60- to 79-year-olds (19%, n=29,492). Although only 12% of cases (n=17,822) occurred among the oldest group (80 years or above), this group was most affected by the disease with an age-specific cumulative incidence of 314 cases per 100,000 inhabitants, followed by 40- to 59-year-olds (226 per 100,000) and 20- to 39-year-olds (209 per 100,000). Infants, children and adolescents were generally less affected with 37 cases per 100,000 among children aged 0 to 4 (1%, n=1,462), and 70 per 100,000 among 5- to 19-year-olds (5.2%, n=8,015). The percentage of cases among older people (aged 60 or older) increased from 16% in reporting week 10 to around 37% in reporting week 15. In contrast, the percentage of cases among 40- to 59-year-olds, which was around 48% in reporting week 11, decreased, and by reporting week 20 had stabilised at around 30%; the same applies to cases among 20- to 39-year-olds, which account for 32% of cases. This same distribution of cases by age over time is also clear from the median age, which increased from 43 years in reporting week 10 to 53 years in reporting week 15, but had decreased again to 46 years by reporting week 20 (Figure 1). Overall, both the median and the mean value are 50 years.

Clinical data was available in 138,464 of the 152,984 cases. Across all age groups, coughs (51%, n=70,099), fever (42%, n=58,447) and general symptoms such as weakness, and muscle and body aches (38%, n=52,025) were most frequently reported. Other frequently reported respiratory symptoms included runny nose (22%, n=30,179) and sore throat (19%, n=26,961). Pneumonia and dyspnoea (shortness of breath) occurred primarily among people aged 60 or older, whereas runny noses were much more common among younger age groups (Table 1). It has been possible to report loss of smell and taste as symptoms since reporting week 17. Between reporting week 17 and reporting week 20, at least one of these symptoms was reported in 9.1% (2,126 of 23,403) of cases.

### 3.2 Disease severity level

As 80% of all cases with clinical data (n=110,789) were not reported to have resulted in hospitalisation, pneumonia, or death, they were assumed to have been mild (Table 2). The percentage of mild cases was highest among younger groups, and fell to 62% among 60- to 79-year-olds and to 38% among patients aged 80 or older. In contrast, the percentage of severe or critical cases was highest among patients aged 80 or older. Almost every second case among this group resulted in a hospitalisation, and one in three cases ended in death. At least one risk factor was specified in 26% (n=12,478) of mild cases (Table 3) and the percentage of cases with underlying conditions increased in line with disease severity – representing 89% of those who died (n=4,223).

#### Severe cases

A total of 18% of cases (n=27,466) were hospitalised, with the highest age-specific proportion among patients aged 80 or older (48%, Table 2). The highest percentage of hospitalised cases occurred in reporting week 16 (22%) and in patients aged under 60 in reporting week 10 (0 to 4 years: 25%; 5 to 19 years: 13%; 20 to 39 years: 13%; 40 to 59 years: 20%). The highest percentage of hospitalised cases among the 60-to-79 age group occurred in reporting week 18 (43%) and for cases aged 80 or older in reporting week 12 (65%). Among men, 21% of cases were hospitalised; whereas this applies to 15% of women (men account for 55% of all hospitalised cases; ratio of men to women=1.2). A total of 23% (n=6,321) of hospitalised cases deceased. The highest numbers of fatal cases in hospital (26%) among severe cases were reported in reporting week 14 (n=1,568) and reporting week 15 (n=1,242).

Information about risk factors was available in 52% of all hospitalised cases (n=14,245). One-third (30%, n=4,228) had no reported risk factor, whereas 17% (n=2,380) had more than three reported risk factors (Table 3). Severe cases among younger groups (0 to 39 years) in particular also presented without any reported risk factors. Overall, many cases that resulted in hospitalisation involved at least one reported risk factor (70%, n=10,017). The most frequently mentioned risk factors were cardiovascular diseases (67%), diabetes (29%) and neurological disorders (29%) (Table 4).

#### Critical cases (intensive care, fatalities)

A total of 24,827 cases that resulted in hospitalisation included data about whether the patient had been treated in ICU. Among these, 14% (n=3,418) received treatment in ICU (Table 2); the majority were men (70%, n=2,396). The largest percentage of cases treated in intensive care was during reporting week 13 (859 cases, 19%). Among all cases treated in ICU that provided information on clinical data (n=3,290) – including, therefore, data about whether the patient had received ventilation – 23% were ventilated. In total, 1,619 (47%) out of 3,418 ICU cases deceased; and among these 523 deceased in ICU (84% out of 626 with information on the relevant dates). Information about reported risk factors for patients treated in ICU was available in 61% of cases (2,071 of 3,418). Among these, 20% had no reported risk factor (n=418), one-third (31%, n=643) had one risk factor and 49% (n=1,010) had at least two risk factors (Table 3). The majority of patients who were treated in ICU had reported risk factors, but age-specific differences became apparent. More than half of 20- to 39-year-olds treated in ICU (53%, n=35) had no reported risk factor. The percentage of patients with no risk factor lowered with increasing age, to 11% (n=54) among those aged 80 or above. The data shows that patients treated in ICU mainly had cardiovascular diseases (70%), followed by diabetes (33%) and neurological disorders (30%, Table 4).

A total of 5.6% (n=8,616) of the cases considered resulted in death (Table 2). More fatalities occurred among men than women (56%, n=4,833). Moreover, most fatal cases were among cases aged 60 or older (95%) and, in particular, 79 or above (63%). Out of the 8,616 fatal cases, 6,321 cases had been hospitalised (73%) and 1,619 had been treated in ICU (this amounts to 26% of fatalities that occurred in hospital). Among hospitalised fatal cases in ICU, and that provided clinical data, 341 had acute respiratory failure (ARDS) and 416 had been ventilated. Information about risk factors was available for 55% (n=4,735) of the fatal cases: around one-third had either one, two or three risk factors, respectively, whereas 11% had no risk factors (Table 3). The most frequently reported risk factors were cardiovascular disease (74%), neurological disorders (37%) and diabetes (30%, Table 4).

### 3.3 Intervals relating to hospitalisation

Intervals relating to hospitalisation were calculated using cases that had only been hospitalised once (n=151,014, 99%) and, depending on the interval in question, that also provided data about symptom onset, hospital admission date and/or death.

#### Interval between symptom onset and hospital admission

In total, 75% of cases were hospitalised within eight days of symptom onset; 50% were hospitalised after four days (Table 5). The interval between symptom onset and hospital admission was one day longer among cases treated in ICU (median: five days), and one day shorter (median: three days) among fatalities. The highest percentage of people admitted to hospital on the day of symptom onset (25%) was found among cases aged o to 4 and among those aged 80 or above. In contrast, the longest interval between symptom onset and hospital admission was identified among 40- to 59-year-olds (median: six days).

#### Length of hospital stay

The cases considered in this study were hospitalised for a median of nine days (Table 5). In total, 75% were discharged after about two weeks at the latest (interquartile range: 4 to 17 days). The median length of hospitalisation was longest among 60- to 79-year-olds at eleven days, followed by ten days among cases aged 80 or older. In addition, the length of hospitalisation increased with disease severity (with the exception of fatalities).

#### Interval between hospital admission and ICU admission

On average, cases that were admitted to hospital were moved directly to ICU (median: zero days); 75% of cases required intensive care no later than three days after hospitalisation, regardless of whether they were ventilated or resulted in a fatality (Table 5). No difference was identified by age group or sex.

#### Length of ICU stay

Of the 3,418 cases that required intensive care, only 723 cases (21%) could be considered (as the majority of cases did not provide dates). On average, cases spent a median of nine days in intensive care (Table 5). The majority (75%) received intensive care for no more than 18 days (interquartile range: 4 to 18 days); men required intensive care slightly longer than women. The longest length of stay in ICU (median: eleven days) was identified among 40- to 59-year-olds, followed by 60- to 79-year-olds (median: ten days). If fatal cases are excluded (n=223, median eleven days, interquartile range: 5 to 21 days), 40-to 59-year-olds still remained for the longest period in ICU with a median of 13 days (interquartile range: 8 to 20), followed by cases aged 80 or older (median twelve days, interquartile range: 4 to 29).

#### Interval between hospital admission and death (in hospital)

A median of nine days elapsed between hospital admission and death in hospital (Table 5); 75% of these cases had died by their 18th day in hospital (interquartile range: 5 to 18 days). On average, this interval was shorter among the very old (median: eight days) and longer among younger groups, men and by increasing severity.

#### Interval between symptom onset and death

A median of eleven days passed between symptom onset and death (Table 5); 75% of fatal cases died within 18 days of symptom onset (interquartile range: 7 to 18 days). On average, this period was one day shorter among cases over 80 (median: ten) and increased with decreasing age. In severe cases that required intensive care, the interval increased to up to 16 days (median).

## 4. Discussion

The aim of this article was to describe disease severity of COVID-19 during the first wave in Germany based on available surveillance data from the national notification system.

### The majority of cases were identified among young and middle-aged adults

The first wave of COVID-19 in Germany was characterised by a high proportion of cases in adults between the ages of 20 and 59 (63%). At the beginning of the outbreak, the affected population was comparatively young, with a median age of 43 years. However, by reporting week 20, the proportion of older cases increased, and the final median age of 50 years over the entire observation period corresponds to the first descriptions of the pandemic in China [11, 12]. With an incidence of 314 cases per 100,000 inhabitants, cases aged 80 or above were most affected. The large number of outbreaks in old people’s and nursing homes at the peak of the wave in spring 2020 may have played a role in this, and would also explain the high incidence in this age group [13]. Buda et al. outline that outbreaks in nursing homes rose continuously between reporting week 13 and reporting week 22, averaging around 19 cases per outbreak. Moreover, these outbreaks led more frequently to transmissions compared with outbreaks in other settings (e.g. households) [13].

### The majority of cases were mild

In total, the first wave of COVID-19 in Germany was primarily characterised by mild cases (80%). However, the proportion of mild cases decreases in the age group 60 years and older and is 38% in the oldest group (80 or older). These findings coincide with the first results from China [14–16], which described old age as the greatest risk factor associated with severe cases and those resulting in death [12, 16, 17]. In addition to a large number of cases among younger people with a comparatively high proportion of mild disease, the extensive testing strategy put in place in Germany for early, precise detection will also have played a role in the high proportion of mild cases. Over time, this strategy was expanded to include contact tracing in outbreaks and the screening of certain population groups so that more mild and asymptomatic cases were covered by the national notification system [18–21]. In addition, increased screening measures put in place due to the high risk in old people’s and nursing homes could have led cases among older people to be identified earlier and, therefore, to be treated more promptly. The short interval between symptom onset and hospital admission in the age group 80 years and older (median: two days) would support this assumption.

### Severe cases were reported predominantly among men aged 60 or older who had at least one risk factor

In total, 18% of cases resulted in hospitalisation and among these 70% had at least one reported risk factor. A comparison of the results of this study with those of the first preliminary analysis of disease severity [10] shows that the proportion of cases with reported risk factors among hospitalised cases has increased from 50% to 70%. However, during the course of the pandemic, reporting of SARS-CoV-2 cases in the notification software became easier, and as such, this increase could be explained by improvements in risk factor reporting. However, risk factors are also distributed differently by age group. Children, adolescents and young adults aged from 20 to 39, in particular, only made up a small proportion of hospitalisations, but were frequently admitted to hospital despite having no reported risk factor. It is important to note that at the beginning of the pandemic, hospitalisation was recommended for all patients who tested positive (including, therefore, for mild cases) in order to ensure isolation and which would explain the high proportion of hospitalisations among younger groups at the beginning of the wave. As the pandemic progressed, almost half of patients aged 80 or older and a third of 60-to 79-year-olds were admitted to hospital. The descriptive analysis also identified sex-specific differences between severe and critical cases: men accounted for 55% of hospitalisations and 70% of all cases treated in ICU. Men also spent one day longer in ICU (median). This is in line with other clinical reports that have also demonstrated more severe cases among men; these can probably be attributed to sex-specific differences in the immune response [22]. However, the evidence is inconclusive and research is still ongoing. The most common risk factors identified were diseases of the cardiovascular system, and diabetes. Compared with the first preliminary analysis of disease severity [10], the proportion of cases with neurological disorders increased among all cases with at least one risk factor. At 25%, these cases were represented as frequently as cases with diabetes. This could be due to the higher percentage of people from old people’s and nursing homes, among whom neurological disorders such as dementia are more common [13, 23, 24]. Diseases of the cardiovascular system and diabetes have also been identified as frequent risk factors in severe cases by other studies in Germany [25–29]. Karagiannidis et al. describe this aspect in detail and lists kidney disease and obesity as other common risk factors [30]. However, obesity cannot be reported systematically by local health authorities in the notification software and kidney diseases are mentioned less frequently in the data, but do seem to play a stronger role, especially among fatal cases [31]. A possible explanation for the differences between the results presented here and Karagiannidis et al. could be the predominant involvement of university hospitals in the latter study [30]. University hospitals specialise in severe cases and treat them disproportionately often due to the therapeutic options that they can provide, such as ECMO (extracorporeal membrane oxygenation) and renal replacement therapies, which are, in turn, beneficial to these cases due to the patents’ risk factors. This would also explain the somewhat lengthier median stay in hospital identified by Karagiannidis et al. [30] (ten days), compared with eight days in Docherty et al. [32] and nine days in the present study. In a first study in Germany, Dreher et al. [33] identified a median of seven days, but used a study population of just 50 cases and did not include any patients who received intensive care. The present analysis suggests a median length of stay in ICU of nine days, which clearly deviates from Tolksdorf et al. (five days) [34]. The present analysis included a larger percentage of fatal cases in the calculation of ICU stay (69%) compared with Tolksdorf etal. [34] (30%). This suggests an overestimation of critical cases in the present analysis due to a higher data completeness among critical cases (cases in ICU, fatal cases), even if these cases tend to be underrepresented in the notification system as a whole (see Chapter 4.1).

### The 40-to-59 age group was hospitalised later and, on average, spent the longest time in intensive care

The length of time between symptom onset and hospital admission is relevant to clinical management. This period was longest among the 40-to-59 age group, at six days (median). At the same time, this age group also demonstrates the longest median ICU stay of eleven days (median total ICU stay nine days). In contrast, the shorter median stay in ICU identified among older cases could be related to the higher proportion of fatalities in older age groups. As such, the length of ICU stay was also calculated after excluding fatal cases. Among cases aged 80 or older, the duration increased from a median of six to twelve days. In contrast, the length of stay among 40- to 59-year-olds increased significantly less (by a median of two days); however, at 13 days (median), this group still had the longest stay in ICU. Since both, the interval between symptom onset and hospitalisation, as well as the length of time spent in intensive care, were longest in this age group, it is possible that the risk of severe cases that this age group faces is underestimated and that these cases are only admitted to hospital at a later and, therefore, more severe stage. As a result, they may require longer periods of hospitalisation and intensive care due to the advanced stage of the disease. However, the small number of cases treated in intensive care that could be considered mean that this analysis can only provide limited findings in this regard, see also the limitations set out below.

### 4.1 Limitations

This analysis primarily faces the limitations typically associated with notification and surveillance data. In line with legal requirements, cases are usually only recorded by the notification system if they have been documented within the health care system by resident doctors, hospitals, laboratories, test centres or additionally by community facilities (e.g. kindergarten, schools). Accordingly, if people with mild or asymptomatic disease do not visit doctors or test centres, they will not be diagnosed and thus not be captured by the notification system. In addition, objectively and subjectively severe cases are more likely to lead to a doctor’s visit and this increases the likelihood of a diagnosis among certain age groups and with increasing severity. As such, mild and asymptomatic cases tend to be underreported whereas severe cases are disproportionately overreported. The proportion of mild cases is consistent with international experience; however, these numbers are also dependent on the recommended and implemented testing strategy as well as testing capacities (all of which had to be established at the beginning of the first wave) and the surveillance system in place (including case definitions and the fact that the analysis only includes laboratory-confirmed cases). It can be assumed that the proportion of mild cases in the present analysis also reflects the comprehensive testing strategy conducted in Germany. In comparison with other studies in Germany, hospitalised cases are well represented in the notification system, but cases requiring intensive care and, thus, ventilation are clearly underrepresented. Data from Tolksdorf et al. [34] and Karagiannidis et al. [30] suggest that around one-third of hospitalisations require intensive care; whereas this study found this to be 12%. A similar picture emerges for cases with existing risk factors. Data completeness for risk factors of 52% among hospitalised cases, who actually seem to be well reported, suggests a general underreporting of risk factors. Furthermore, the German notification system can only provide a rough distribution of risk factors and therefore can only provide direction for further research. In addition, the high proportion of fatal cases among the cases treated in ICU suggests that information on ICU stay are better reported for fatal cases (or cases with a long period of hospitalisation) in the notification system, but at the same time are also overestimated.

Finally, once cases have been reported and recorded, it is difficult for local health authorities to follow up on them and to gain information about changes over time and report this updated information afterwards. This can mean that precise information about disease severity, in particular, is delayed or unavailable. However, if further information is available, it is usually available for severe and critical cases.

### 4.2 Conclusion

During the first COVID-19 wave in Germany, the majority of cases were mild. The high proportion of severe cases among people aged 60 or older confirms initial findings about the correlation between disease severity and increasing age. The data on 40- to 59-year-olds, and this particularly applies to critical cases, could indicate that the risk of severe disease increases earlier than previously thought; this should be further investigated. Cardiovascular diseases, diabetes and neurological disorders were reported as relevant risk factors. Due to the limitations of the surveillance data used here, however, it is not possible to determine causal relations between risk factors and disease severity. Nevertheless, this analysis provides valuable information about disease severity of COVID-19 and particularly affected groups.

## Key statements

- The majority of COVID-19 cases reported during the first wave were among young or middle-aged adults.
- Most cases were mild.
- Severe cases were reported predominantly among men aged 60 or older who had at least one risk factor.
- The 40-to-59 age group was hospitalised at a later stage than other age groups and, on average, spent the longest time in intensive care.

## Figures and Tables

**Table 1:** Age-specific percentage of reported symptoms in cases with clinical data (n=138,464, multiple responses possible)* Source: COVID-19 cases reported to the RKI (data as of 26 October 2020, 00:00)

Symptom	0–4 years	5–19 years	20–39 years	40–59 years	60–79 years	≥80 years	Total
N (clinical data available)	1,251	7,012	39,023	49,451	26,788	14,939	138,464
N (number of responses)	1,835	11,230	76,902	102,149	52,097	23,213	267,426
**%**		**%**	**%**	**%**	**%**	**%**	**%**
Coughs	40	42	52	57	50	33	51
Fever	48	34	39	45	45	40	42
General symptoms	18	30	38	40	39	33	38
Runny nose	23	25	29	24	16	6.9	22
Sore throat	8.5	21	25	22	14	5.1	19
Dyspnoea	2.2	3.2	7.1	8.3	13	16	9.3
Diarrhoea	6.5	4.7	6.2	7.9	9.0	6.6	7.3
Pneumonia	0.6	0.2	0.7	2.0	7.0	11	3.5
ARDS	0.1	0.1	0.2	0.6	2.1	2.5	1.0
Tachycardia	0.1	0.0	0.1	0.2	0.5	0.5	0.2
Tachypnoea	0.1	0.0	0.1	0.1	0.1	0.2	0.1

*It has only been possible to report loss of smell and taste since reporting week 17; these data are not shown here.

ARDS = Acute Respiratory Distress Syndrome

**Table 2:** Age-specific distribution according to disease severity level (n=152,984 cases). Data on mild and moderate courses refer to cases with clinical information (n=138,464) Source: COVID-19 cases reported to the RKI (data as of 26 October 2020, 00:00)

Total number of cases with information on disease severity level (yes, no)		Number of cases by disease severity level	Percentage (%) of all cases in this age group by disease severity level
**Mild (no pneumonia, no hospitalisation, not deceased)**			
**Total**	**138,464**	**110,789**	**80**
0–4 years	1,251	1,099	88
5–19 years	7,012	6,772	97
20–39 years	39,023	36,940	95
40–59 years	49,451	43,777	89
60–79 years	26,788	16,488	62
≥80 years	14,939	5,713	38
**Moderate (pneumonia, no hospitalisation, not deceased)**			
**Total**	**138,464**	**442**	**0.3**
0–4 years	1,251	1	0.1
5–19 years	7,012	11	0.2
20–39 years	39,023	101	0.3
40–59 years	49,451	195	0.4
60–79 years	26,788	101	0.4
≥80 years	14,939	33	0.2
**Severe (hospitalisation, irrespective of intensive care and death)**			
**Total**	**152,984**	**27,466**	**18**
0–4 years	1,462	165	11
5–19 years	8,015	256	3.2
20–39 years	42,801	2,154	5.0
40–59 years	53,392	5,725	11
60–79 years	29,492	10,628	36
≥80 years	17,822	8,538	48
**Intensive care (hospitalisation, and treated in intensive care)**			
**Total**	**24,827**	**3,418**	**14**
0–4 years	142	**7**	4.9
5–19 years	199	4	2.0
20–39 years	1,769	99	5.6
40–59 years	5,037	695	14
60–79 years	9,746	1,833	19
≥80 years	7,934	780	10
**Fatalities (irrespective of symptoms and hospitalisation)**			
**Total**	**152,984**	**8,616**	**5.6**
0–4 years	1,462	1	0.1
5–19 years	8,015	1	0.0
20–39 years	42,801	31	0.1
40–59 years	53,392	374	0.7
60–79 years	29,492	2,819	9.6
≥80 years	17,822	5,390	30

**Table 3:** Age-specific distribution of cases with data on risk factors (n=65,872) Source: COVID-19 cases reported to the RKI (data as of 26 October 2020, 00:00)

Total number of cases with information on risk factors (yes, no)		No risk factor (%)	One risk factor (%)	Two risk factors (%)	Three or more risk factors (%)
**Mild (no pneumonia, no hospitalisation, not deceased)**					
**Total**	**47,767**	**35,289(74)**	**8,282(17)**	**3,092 (6.5)**	**1,104 (2.3)**
0–4 years	460	429(93)	22 (4.8)	6 (1.3)	3 (0.7)
5–19 years	2,865	2,673(93)	172 (6.0)	17 (0.6)	3 (0.1)
20–39 years	15,780	14,097(89)	1,426 (9.0)	228 (1.4)	29 (0.2)
40–59 years	18,781	13,817(74)	3,636(19)	1,069 (5.7)	259 (1.4)
60–79 years	7,390	3,675(50)	2,210(30)	1,116(15)	389 (5.3)
≥80 years	2,491	598(24)	816(33)	656(26)	421(17)
**Moderate (pneumonia, no hospitalisation, not deceased)**					
**Total**	**174**	**104(60)**	**41(24)**	**25(14)**	**4 (2.3)**
0–4 years	1	1(100)	0 (0)	0 (0)	0 (0)
5–19 years	6	6(100)	0 (0)	0 (0)	0 (0)
20–39 years	40	32(80)	7(18)	0 (0)	1 (2.5)
40–59 years	81	49(60)	23(28)	8 (9.9)	1 (1.2)
60–79 years	34	12(35)	10(29)	11(32)	1 (2.9)
≥80 years	12	4(33)	1 (8.3)	6(50)	1 (8.3)
**Severe (hospitalisation, irrespective of intensive care and death)**					
**Total**	**14,245**	**4,228(30)**	**4,500(32)**	**3,137(22)**	**2,380(17)**
0–4 years	68	58(85)	5 (7.4)	5 (7.4)	0 (0)
5–19 years	119	86(72)	19(16)	11 (9.2)	3 (2.5)
20–39 years	989	730(74)	192(19)	55 (5.6)	12 (1.2)
40–59 years	2,822	1,418(50)	847(30)	377(13)	180 (6.4)
60–79 years	5,644	1,226(22)	1,969(35)	1,388(25)	1,061(19)
≥80 years	4,603	710(15)	1,468(32)	1,301(28)	1,124(24)
**Intensive care (hospitalisation, and treated in intensive care]**					
**Total**	**2,071**	**418(20)**	**643(31)**	**516(25)**	**494(24)**
0–4 years	2	1(50)	1(50)	0 (0)	0 (0)
5–19 years	2	0 (0)	0 (0)	1(50)	1(50)
20–39 years	66	35(53)	19(29)	10(15)	2 (3.0)
40–59 years	396	151(38)	128(32)	65(16)	52(13)
60–79 years	1,099	177(16)	358(33)	290(26)	274(25)
≥80 years	506	54(11)	137(27)	150(30)	165(33)
**Fatalities (irrespective of symptoms and hospitalisation)**					
**Total**	**4,735**	**512(11)**	**1,428(30)**	**1,436(30)**	**1,359(29)**
0–4 years	0	0 (0)	0 (0)	0 (0)	0 (0)
5–19 years	1	0 (0)	1(100)	0 (0)	0 (0)
20–39 years	17	5(29)	7(41)	5(29)	0 (0)
40–59 years	224	47(21)	81(36)	47(21)	49(22)
60–79 years	1,575	174(11)	498(32)	440(28)	463(29)
≥80 years	2,918	286 (9.8)	841(29)	944(32)	847(29)

**Table 4:** Distribution of reported risk factors in severe cases (n=24,085, multiple responses possible) Source: COVID-19 cases reported to the RKI (data as of 26 October 2020, 00:00)

Risk factors	Total		Hospitalised		Intensive care unit		Fatalities	
n		%	n	%	n	%	n	%
**Cases with at least one risk factor**	**24,085**		**10,017**		**1,653**		**4,223**	
Cardiovascular diseases	14,816	62	6,682	67	1,156	70	3,125	74
Neurological disorders	6,119	25	2,893	29	494	30	1,543	37
Diabetes	5,649	24	2,863	29	549	33	1,275	30
Lung diseases	5,309	22	2,171	22	418	25	914	22
Kidney disease	2,725	11	1,647	16	316	19	978	23
Immunological disorders	2,360	9,8	1,051	11	172	10	418	9.9
Cancer	2,425	10	1,297	13	218	13	620	15
Liver disease	683	2.8	352	3.5	75	4.5	160	3.8

**Table 5:** Intervals (in days) related to hospitalisation and death by age group, sex, intensive care status, ventilation status and death Source: COVID-19 cases reported to the RKI (data as of 26 October 2020, 00:00)

Symptom onset until hospital admission			Length of hospital stay		Hospital admission to ICU admission		Length of ICU stay		Hospital admission until death in hospital		Symptom onset until death	
**n**		**Median d (IQA)**	**n**	**Median d (IQA)**	**n**	**Median d (IQA)**	**n**	**Median d (IQA)**	**n**	**Median d (IQA)**	**n**	**Median d (IQA)**
**Total**	14,043	4 (1-8)	11,504	9 (4-17)	723	0 (0-3)	723	9 (4-18)	4,532	9 (5-18)	5,888	11 (7-18)
**Age groups** 0–4 years 5–19 years 20–39 years 40–59 years 60–79 Jahre ≥80 years	78 97 1023 3,325 5,463 4,057	1 (0–3) 3 (1–7) 5 (2–9) 6 (3–9) 4 (1–8) 2 (0–6)	67 72 685 2,165 4,302 4,213	2 (1–4) 2 (1–5) 4 (2–9) 7 (4–12) 11 (6–19) 10 (5–18)	1 0 15 108 393 206	0(0–0) N.A. 0 (0–1) 0 (0–2) 0 (0–3) 0 (0–4)	1 0 15 108 393 206	5 (5–5) N.A. 5 (2–13) 11 (6–21) 10 (4–19) 6 (3–11)	0 1 15 216 1,686 2,614	N.A. 38 (38–38) 16 (10–22) 15 (7–26) 12 (6–21) 8 (4–15)	0 1 21 264 1,931 3,671	N.A. 37 (37–37) 20 (11–29) 17 (9–28) 14 (8–23) 10 (6–16)
**Sex** Women Men	6,026 8,011	4 (1–8) 4 (1–8)	5,038 6,416	9 (4–16) 9(5–17)	206 517	0 (0–3) 0 (0–3)	206 517	8 (3–15) 9 (4–18)	1,745 2,786	8 (4–16) 10 (5–19)	2,532 3,355	10 (6–16) 12 (7–20)
**Intensive care unit** Yes No	2,093 11,950	5 (1–8) 4 (1–8)	1,762 9,742	14 (7–25) 8 (4–15)					1,200 3,332	11 (6–21) 9 (4–16)	1,174 3,014	16 (9–25) 11(7–17)
**Ventilation** Yes No	696 13,347	4 (1–8) 4 (1–8)	609 10,895	16 (7–29) 9 (4–16)	161 562	0 (0–3) 0 (0–3)	161 562	12 (6–20) 8 (3–17)	437 4,095	12 (6–23) 9 (4–17)	456 5,432	16 (9–27) 11 (6–17)
**Fatalities** Yes No	3,337 10,706	3 (0–6) 5 (1–9)	4,709 6,795	9 (5–18) 9 (4–16)	500 223	0 (0–3) 0 (0–3)	500 223	8 (3–16) 11 (5–21)				

*Empty cells: no differentiation was made by ICU status for intervals relating to the length of a stay in ICU, because they already include all cases that involved a stay in ICU. For intervals calculated using the date of death, no differentiation was made for fatal cases because these cases are already included in the calculation.

Any discrepancies between individual categories and the total number of cases are due to missing information.

d = days, ICU = intensive care unit, IQR = interquartile range, N.A. = no data
